# Blue Biotechnology: Marine Bacteria Bioproducts

**DOI:** 10.3390/microorganisms12040697

**Published:** 2024-03-29

**Authors:** Karina Maldonado-Ruiz, Ruth Pedroza-Islas, Lorena Pedraza-Segura

**Affiliations:** Department of Chemical, Industrial and Food Engineering, Universidad Iberoamericana, Prol. Paseo de la Reforma 880, Lomas de Santa Fe, Mexico City 01210, Mexico; p36647@correo.uia.mx (K.M.-R.); lorena.pedraza@ibero.mx (L.P.-S.)

**Keywords:** bacteria, marine, enzymes, bacteriocin, exopolysaccharides

## Abstract

The ocean is the habitat of a great number of organisms with different characteristics. Compared to terrestrial microorganisms, marine microorganisms also represent a vast and largely unexplored reservoir of bioactive compounds with diverse industrial applications like terrestrial microorganisms. This review examines the properties and potential applications of products derived from marine microorganisms, including bacteriocins, enzymes, exopolysaccharides, and pigments, juxtaposing them in some cases against their terrestrial counterparts. We discuss the distinct characteristics that set marine-derived products apart, including enhanced stability and unique structural features such as the amount of uronic acid and sulfate groups in exopolysaccharides. Further, we explore the uses of these marine-derived compounds across various industries, ranging from food and pharmaceuticals to cosmetics and biotechnology. This review also presents a broad description of biotechnologically important compounds produced by bacteria isolated from marine environments, some of them with different qualities compared to their terrestrial counterparts.

## 1. Introduction

Blue or marine biotechnology has been an emerging area since 1940, when the search began for microorganisms and enzymes with different characteristics that will give them an advantage in industrial use [1]. For example, these characteristics include the ability to withstand high or low temperatures and high salt concentrations, tolerance to high pressures, or changes in the membranes, such as an increase in their fluidity caused by the presence of negatively charged phospholipids, and the ability to produce certain compounds such as docosahexaenoic acid (DHA) and eicosapentaenoic acid (EPA) [2]. Marine microorganisms are usually more enzymatically active than their terrestrial counterparts, due to their phylogenetic differences and their niche [3,4]. On the other hand, the nutrition of these microorganisms has a particular context because some compounds are dissolved, and most of the carbon sources are in the form of complex molecules. In order to access these substrates, special enzymes which are not synthesized all the time are required to break down the molecules. To produce and excrete these enzymes, the microorganism first needs to adhere to the substrate, and after that, it finally excretes the enzymes [5].

**Figure 1:**
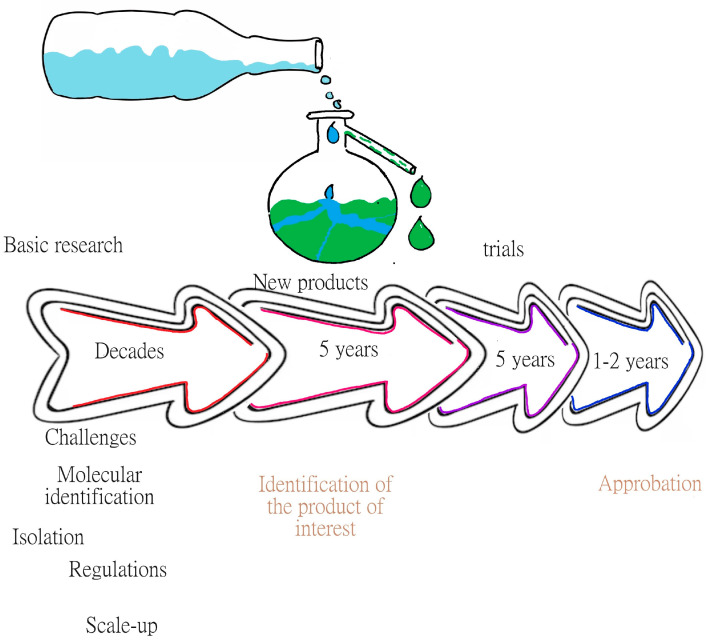
Line of discovery of products of marine origin. The figure shows that the most difficult part of the discovery is in the early years, where different challenges are faced, such as identifying and cultivating microorganisms and establishing the necessary regulations, among others.

Currently, 600 new species of marine microorganisms are identified and cataloged every year, and it would take up to 1 million years to catalog all the existing species, since it is assumed that there are 2.2 ± 0.18 million marine species [6], which is a restrictive number. It is also assumed that there are prokaryotes in at least ~1.3 million species in the world, of which approximately half have been cataloged, notwithstanding the characterization and identification of the products that may become of biotechnological interest. These data explain, in part, the time invested in the development of products of microbial origin. For example, Pharma Mar S.A. (Madrid, Spain, E.U.) discovered a drug for cancer treatment which then took 15 to 20 years from discovery to commercialization, and while the cost depends on the processes, for that company, it was 802 million USD, resulting in 5 molecules that worked but only 2 molecules were used to recover the investment costs. Figure 1 shows the path of discovery of new products of marine origin. Further, due to their biological activities, the marine environment has proved to be a good source of microorganisms that produce novel compounds with great potential for industrial application [7].

This review shows some of the metabolites of interest produced by marine microorganisms, such as bacteriocins, pigments, exopolysaccharides, and enzymes, which are molecules of great interest and industrial application.

## 2. Bacteriocins 

Bacteriocins are among the metabolites that bacteria produce as defense mechanisms. They are peptides that and can inhibit the growth of some bacteria of related species [5], and even other species that are phylogenetically distant [8]. Due to the diversity of their structures, molecular weights, and properties, various classifications for bacteriocins have been suggested, among which stands out the classification that organizes them into three classes, depending on their biosynthesis mechanisms and their biological properties. Class I has extensive post-translational peptides of 19 to 50 amino acids, which includes lantibiotics such as nisin, which is the best known; Class II has non-lantibiotics, comprising unmodified small peptides with molecular weights <10 kDa that arethermostable andhydrophobic, such as pediocin-like bacteriocins; while Class III has peptides of greater weight, >30 kDa, that are thermolabile andhydrophilic such as for example helveticin [8]. 

Another widely-accepted classification is based on the type of cell wall of the bacteria that produce bacteriocins, i.e., gram-negative and gram-positive bacteria. The bacteriocins produced by the former have been organized into four classes: (1) colicins, which have been the most studied; (2) colicin-like bacteriocins; (3) phage tail-like bacteriocins; and (4) microcins [9]. Regardless of the classification, the bacteriocins produced by gram-negative bacteria come mainly from microorganisms of the Enterobacteriaceae family. Another widely-accepted classification is based on the type of cell wall of the bacteria that produce them: gram-negative and gram-positive bacteria. The bacteriocins produced by the former have been organized into four classes: colicins, which have been the most studied; colicin-like bacteriocins; phage tail-like bacteriocins; and microcins [9]. 

Bacteriocins from gram-positive bacteria have been classified into three classes: Class I, comprising <10 kDa, positively charged, linear A-type peptides including lantibiotics characterized by their extensive post-translational modifications; Class II, comprising peptides of low molecular weight <10 kDa, without post-translational modification; and Class III, comprising peptides of >10 kDa, that are thermolabile and can exhibit bacteriolytic and non-lytic mechanisms of action [10]. The bacteriocins produced by this group of bacteria are the best known, the most abundant, and the most successfully used, with the advantage that their inhibitory capacity is not only for related bacteria but also has a broader spectrum [9]. They are also the largest source examined for biotechnological applications [11], with the most studied being bacteriocins that come from lactic acid bacteria, generally recognized as safe (GRAS) or have qualified presumption of safety (QPS), being isolated from milk and fermented milk products [12]. 

The mechanisms of action are varied, such as those that exert a function on the cell wall, those that inhibit lipid II at the membrane level, canceling the synthesis of peptidoglycan, and others that are via the formation of pores or by interference with RNA and DNA and protein metabolism. These mechanisms have been described in various publications [8]. The current interest and relevance of bacteriocins are due to the fact that they can be an effective alternative to traditional antibiotics, with low toxicity, with the clinical application against pathogenic bacteria resistant to antibiotics [13,14,15] and, since practically all bacteria can produce bacteriocins [16], the variety of these metabolites is so great that it offers the possibility of having bacteriocins for each specific pathogen of interest in humans, and even in animals. 

Different ecosystems have been explored, such as deep waters, where conditions are extreme due to the absence of light, high hydrostatic pressures, low temperatures, and scarcity of nutrients, finding bacteria with a diverse genetic reservoir, which makes them sources of new products, among which bacteriocins are found [17]. Genetic mappings have also been made in brine pools, determining that 0.464% of all the genes found correspond to bacteriocins [18]. 

Some of the marine bacteria studied for the production of bacteriocins, for which the type of bacteriocin, its antagonistic activity, and its molecular weight are known, are presented in Table 1. 

Other examples of bacteriocin-producing gram-positive marine bacteria are: *Bacillus* sp. isolated from a marine sponge and capable of producing subtiliomycin [21,22]; *Carnobacterium divergens* isolated from mussels and producer of divergicin M35, which seems to have different characteristics such as production at low temperatures; *Staphylococcus hominis* KQU-131 obtained from fermentation of a marine fish [23], *Carnobacterium alterfunditum* from Antarctica [24], and *Lactobacillus lactis* from olive sole and perch [25,26], *Bacillus licheniformis*, isolated from marine algae, with linchenicin-producing activity against Gram+ and Gram- bacteria, *Bacillus pumilus* and *Lactococcus lactis* from green algae [27], *Lactobacillus casei* obtained from sardines [28], *Bacillus pumilus* and *Lactobacillus plantarum* from lobster [29]. 

Gram-negative marine bacteria include *Vibrio anguillarum*, isolated from the intestines of healthy fish, which produces vibriocin [30], *Marinomonas piezotolerans* from marine sediment in Japan, *Streptomyces* sp. from a marine sponge [27], *Marinomonas meditrranea* [31], among others. 

To facilitate the finding of bacteriocins, some authors have suggested the use of bioinformatic tools such as the BADASS v1.2 (BActeriocin-Diversity ASsessment Software) database, which helps to identify bacteriocins [32].

Bacteriocins are also an option for the increasing demand for natural preservatives for food products [33]. Nisin, produced by *Lactococcus lactis* sbsp. *lactis*, a gram-positive lactic acid bacterium, widely used in fermentation processes [34], is the first bacteriocin approved for use in cheese spreads to prevent the growth of *Clostridium botulinum* [35] and its use is currently permitted in other foods such as dairy products, meat products, bakery products, etc.; while another permitted bacteriocin is natamycin which is produced by various *Streptomyces* species [36]. 

For the approval of bacteriocins for use in food, certain criteria must be met, e.g., that the producing strains are food grade (GRAS), have a broad spectrum of inhibition, have high specific activity, have no risks associated with health, and if possible, present beneficial effects on the safety, quality, and flavor of food or simply do not alter any characteristic of the food, have pH stability and solubility [37]. Its application for food preservation can be by inoculation with the bacteriocin-producing strain, addition of purified or semi-purified bacteriocin as a food additive, and use of a previously fermented product with a bacteriocin-producing strain as an ingredient in food. It is noteworthy that the effectiveness of bacteriocins to inactivate pathogens, which may be in food, may differ depending on the food matrix in question, which implies that they should be tested in all food systems. On the other hand, in most cases, bacteriocins are adsorbed in food matrices and are easily degraded, resulting in a loss of antibacterial activity [38]; an alternative is its incorporation into coatings [39]. 

Another relevant use of bacteriocins is in aquaculture, where it has been seen to help the immune system of fish and shrimp [17] since bacteriocin-producing marine bacteria occupy the same ecological niche as marine pathogens in fish, taking into account that the use of terrestrial microorganisms was not functional [21]. 

Further, among the most important challenges to solve is the need to increase the production yield of bacteriocins, reduce purification costs, and make it profitable at the industrial level [40].

## 3. Pigments

The use of bacterial pigments of marine origin has grown in recent years because they are considered “natural” compounds, which are environmentally safe, and beneficial for health. Natural pigments are a group of chemically heterogeneous molecules found in animals, plants, and microorganisms and are useful as camouflage, attracting partners, and as a warning system, among others. Due to the remarkable chemistry of marine organisms, many species display a wide range of colors, many of which display various biological properties and constitute an adaptive evolutionary element [41]. Table 2 shows some of the main pigments produced by marine bacteria. Pigments such as astaxanthin, zeaxanthin, fucoxanthin, neoxanthin, lutein, and violaxanthin, which belong to a group of phytochemicals, carotenoids, are polyunsaturated hydrocarbons containing 40 carbon atoms per molecule, are orange and red in color, and are commonly produced by plants, but some exceptions have been found, such as *Flavobacterium* [41]. Within the carotenoids are carotenes and xanthophylls; the former are hydrocarbons and the latter are oxygenated derivatives. One of the most commercially valuable carotenoids is astaxanthin, which can be obtained from marine microorganisms and is used as an additive to animal feed, and as a food supplement for humans, and some that produce microorganisms are *Agrobacterium aurantiacum* and *Altererythrobacter ishigakiens* [42]. Studies on the possible uses of astaxanthin are diverse, but the most notorious are those found in the medical field since there is talk of its potential as an antioxidant and its anti-inflammatory activity, because it exerts protective effects on liver cells after the induction of inflammation [43], and protects neuronal cells from oxidative stress through the activation of specific pathways [44]. Recent studies have demonstrated the beneficial effects of carotenoids for the treatment of neurodegenerative diseases, while several epidemiological studies have linked the consumption of a diet rich in carotenoids with a reduced risk of neurodegenerative diseases in humans [45]. Tambjamins have been found in marine microorganisms and exhibit broad-spectrum microbial activity; they have an alkaloid molecule that can also be found in some organisms such as bryozoans [46]. Quinones have an aromatic ring in their structure and their color ranges from yellow to red, depending on the molecule. Quinones work as antiviral, antifungal, antibacterial, antitumor, and commercially as dyes due to their stability. Chinikomycin A and B have recently been shown to have anticancer activity for melanoma, breast, and kidney cancer [47]. Prodigiosins are red pigments, tripyroles with a system of rings connected by a methane bridge. They exist in isomeric forms, and the microorganisms reported as producers are found in Table 2.

### 3.1. Biosynthesis and Industrial Uses 

Pigment biosynthesis has been extensively studied; however, only a few of its mechanisms are known, and it is not known with certainty whether all pigments use similar pathways for their production.

#### 3.1.1. Carotenoid Biosynthesis

To date, around 700 marine and terrestrial carotenoids have been identified, among which are lutein, astaxanthin, lycopene, and beta-carotene, among others. The synthesis of carotenoids involves several intermediaries, e.g., lycopene, which is a colorless intermediate that is modified to produce different carotenoids; beta carotene is generated by the cyclization of lycopene by an enzyme called lycopene beta cyclase, and from there, it can be converted to canthaxanthin through the catalytic activity of the enzymes beta carotene ketolase and beta carotene hydroxylase [60].

The groups of genes that have a role in the synthesis of carotenoids are different for each species. In the bacterium *Brevundimonas* sp. SD212, 2-hydroxyastaxanthin is synthesized with the *crtW*, *crtY*, *crtI*, *crtB*, *crtE*, *id*i, and *crtZ* genes; in *Agrobacterium aurantiacum* the genes used are crtW, crtZ, crtY, crtI, and *crtB*, which shows that in both species 70% of the genes are shared. However, in the case of *Brevundimonas* sp., more genes are responsible for the synthesis [61].

#### 3.1.2. Prodigiosin Biosynthesis 

Prodigiosin biosynthesis occurs in two stages, as in other secondary metabolites. The first phase is called trophophase, which is the nutrition phase in which cell growth occurs, and the second phase is idiophase, which is when secondary metabolites are produced [62], appearing in the last stages of microbial growth, and they seem to be influenced by environmental factors such as temperature, pH, etc. Prodigiosin biosynthesis is controlled by a dependent and independent N-acetyl homoserine lactone quorum sensing, and this reaction is related to temperature. The precursors can be serine, alanine, methionine, and alanine. It has 15 biosynthesis genes [63].

#### 3.1.3. Melanin Biosynthesis 

Melanins are heterogeneous pigments formed by oxidative polymerization of indole or phenolic compounds. They are formed through the oxidation of hydroxylated aromatic compounds that give rise to reactive quinones, which polymerize to form heterogeneous pigments in the presence of oxygen. Most bacterial melanins are formed due to transformations of aromatic amino acids such as tyrosine [64], and some bacteria, such as *Streptomyces*, can produce melanin from malonyl-CoA in a process catalyzed by polyketide synthases [65].

#### 3.1.4. Tambjamin Biosynthesis 

Tambjamins are yellow-pigmented alkaloids with a conserved bi-pyrrole nucleus and a functionalized imine residue; often the imine is an amino acid side chain. The genes involved in the biosynthesis of tambjamins (*Tam*) are known as the *Tam* metabolic pathway. The genes are *Tam A*, with two domains, one of which is the adenylation domain that adenylates the fatty acid, *Tam T* is a dinucleotide adenine flavin suspected to introduce the double bond to the alkyl chain, *Tam H* has two active sites, a thiol reductase and an aminotransferase, while *Tam* C is an oxidase responsible for cyclization [59].

#### 3.1.5. Indigoid Biosynthesis 

Indigo pigments are widely used in the textile, food, and medical industries, and numerous microorganisms capable of producing indigo pigment have been isolated and characterized. Two enzymes have been identified in this biosynthesis: monooxygenase and dioxygenase; the latter activates and catalyzes the addition of one or two oxygens to aromatic substrates, and the former can be classified into two types, monochrome monooxygenase and flavin monooxygenase. Both types can catalyze the addition of molecular oxygen to aromatic substrates [66].

#### 3.1.6. Violacein Biosynthesis 

Violacein is a purple pigment with antitumor and antimicrobial activities, its biosynthesis begins with the oxidation of a tryptophan precursor molecule to convert it into indole-3-pyruvic acid by the *Vio A* flavoenzyme; subsequently, *Vio B* couples two imine dimer molecules, and the enzyme *Vio E* converts it to protodeoxyviolacein acid, Vio D forms protoviolacein acid, and *Vio C* hydroxylates the molecule to form violacein [66].

#### 3.1.7. Phenazines Biosynthesis 

They are redox-active pigments that contain aromatic compounds, are closely related to the quorum sensing of bacteria, and have many industrial uses ranging from antibiotics, insecticides, antivirals, etc. Their biosynthesis is carried out by means of five enzymes, *Phz. Phz E* converts chorismic acid to 2-amino-2-deoxychorismic acid, *Phz D* converts chorismate to DHHA< (Trans-2,3-dihydro-3-hydroxyanthranilic acid), *PHzF* isomerizes, *Phz B* produces HHPDC (Hexahydrophenazine-1, 6-dicarboxylic acid) which is unstable and undergoes decarboxylation, and *Phz G* produces the central and final phenazine compounds [58].

The role of these secondary metabolites is not known with certainty for all cases, but it is assumed that their function is protection for the producing bacteria against other bacteria found in the environment. These activities have great potential in the food, pharmaceutical, and agricultural industries, since their applications range from medicines and biopesticides, to “natural” food preservatives, or dyes in the textile industry due to their good stability [61].

The role of pigments in the microbial life cycle depends on the situation of the microorganism (ecosystem, niche), e.g., Salikin et al. [67] reported that, when comparing marine and terrestrial strains, the former produces a pigment (phenazines) that is active against marine larvae and is synthesized when the bacterium adheres to a surface, acting as an adaptation and protection mechanism against predators. Regarding pigments such as melanins and acytonemins, their biological function is an adaptation of bacteria to avoid damage from UV rays and desiccation. Shanta et al. [68] reported that pigment-producing bacteria have greater resistance to metals and antibiotics, also being a form of adaptation. Likewise, there are other pigments reported in the same way, referring to adaptations of marine microorganisms due to the lack of iron in the marine environment [69].

Pigments of marine origin are already used in different nutritional supplements, antibiotics, skincare, and other applications [59]. The pigments with the greatest industrial value are beta carotene, lutein, and, as aforementioned, astaxanthin, since their antioxidant properties are important for use in the food and cosmetic industries. An astaxanthin market is estimated to have sales of around 3.4 billion USD with a growth rate of 16.2% for the year 2027. In the case of carotenoids, it has been estimated that the market grew by 5.7% up to 2022 [59,70].

## 4. Exopolysaccharides

Polysaccharides are important constituents as they provide structure to plant cell walls (cellulose), are storage polymers (starch), or fulfill various functions, such as exopolysaccharides (EPSs) obtained from microorganisms.

EPSs are extracellular polysaccharides produced by bacteria with various functions such as adherence, metal adsorption, primary cell maintenance, antibacterial activity, viral activity, and protection against predators, among others. Hence, their function will be given by the ecological niche of the organism that produces them [30,71,72,73]. Their potential applications are in the paper, food, textile, pharmaceutical, cosmetic, and mining industries, among others, which is why today they are presented as an ecological substitute for polymers of fossil origin [74,75,76].

The classification of EPSs can be very varied but can be simplified into homopolysaccharides and heteropolysaccharides, depending on their composition (if they have a single type of monosaccharide or several types), and in turn, they can be divided into linear or branched. Table 3 shows some components [30,77].

Microorganisms can produce more than one EPS at the same time or several at different stages, e.g., *Pseudomonas* sp. produces two different EPSs, one contains glucose and the other galactose; the former is produced in the exponential growth phase and the latter during the stationary phase [78]. As with this case, there are many other cases where the production of EPSs will depend on the components that the microorganism has in the medium, pH, temperature, salinity, growth phase, and stress factors [79].

Under natural conditions, a bacterium can produce 1 g/L of EPSs, but under controlled conditions (laboratory) a bacteria can produce more than 50 g/L [80]. Low yields in nature are because EPS production requires 70% of cellular energy, e.g., in some cases, such as with *Halomonas alkaliantarctica* [81,82,83]. The production of EPSs is in competition with the formation of cell membrane components due to the metabolic pathways used to produce both compounds and their raw materials [74,84].

The advantages of EPSs produced by bacteria are: higher production volume, they come from renewable sources, their production can be controlled by culture conditions, production times are shorter compared to plants, conditions can be more controlled and can be carried out with good manufacturing practices, with defined media, GRAS microorganisms, they do not depend on the climate, and their recovery is simply by using ethanol [85,86,87,88,89]. Table 4 shows some EPS-producing marine microorganisms and their possible applications.

Among the potential uses of marine EPSs is the use as prebiotics since they help to regulate the intestinal microbiome by functioning as an energy source and allowing homeostasis to be maintained. Studies have been carried out in mice with *Gelidium pacificum* and *G. cereus*, where positive results were obtained, such as a decrease in intestinal inflammation, an increase in good intestinal microbiota for mice, and a decrease or elimination of harmful microbiota [72].

Within the medical field, EPSs have been used for treatments against arthritis [77], obesity, and colitis [72], as antivirals, antibacterials [84], and antioxidants, and some against certain types of cancer such as breast cancer, where it has been shown that they increase apoptosis in cancer cells [89].

In their use as antioxidants, good results have been obtained in EPSs with a size of 10 to 100 kDa [77], which is achieved thanks to the fact that they have “reducing ends” that are used to react with free radicals [86].

Another use for EPSs in medicine, food, and microbiology is for cryopreservation, which is why EPSs of marine microorganisms from cold areas are sought [94] because they serve to protect microorganisms from the cold. When EPSs are compared with the use of glycerol for cryopreservation, similar results are obtained for the number of living cells, but an improvement is shown in the case of the number of freezing cycles (number of times the sample is frozen and thawed) in tests performed on *E. coli*, where significant damage to their cellular lipids was shown with the use of glycerol, and there was less damage with the use of EPSs, revealing their role as protectors [95].

EPSs have various routes for their production, among which three stand out and are the most studied:

The first pathway is that of the *WZx* and *WZy* genes, and it occurs in the cytoplasm with membrane proteins, and the steps are (1) introduction of sugars into the cell, phosphorylation with phosphoglycosyl transferase, (2) polymerization that occurs in the periplasmic space via the *Wzy* protein before they are exported to the cell surface, and (3) the transport of the polymerized repeat units from the periplasm to the cell surface, which is dependent on additional proteins assigned to the copolymerase polysaccharide families. All polysaccharides assembled by the *WZx* and *WZy* have a highly diverse sugar pattern (five types of sugar within their chemical structure), therefore microorganisms that use this pathway carrying the flippase (*WZx*) and polymerase (*WZy*) genes are classified as heteropolymers [71,74].

The second pathway is *ABC*, which is somewhat similar to *WZx* and *WZy* with the difference that, instead of being carried out in the cytoplasm it is carried out in the periplasm. The EPSs are formed by the action of glycosyl transferase on the cytoplasmic side of the inner membrane, resulting in homopolymers when only one type of glycosyltransferase is used. But when multiple types are used for the assembly process there are heteropolymers, which export across the membrane and out to the cell surface. This is a different pathway because it is carried out by a tripartite flow pump that is composed of ABC transporters which cross the inner membrane and secrete the polymer completely with the help of a lipid that is present in the structure [71,80].

The third pathway is outside the cell where the EPSs can be produced with glycosyltransferases which synthesize them completely through the membranes and the cell wall. The polymerization and the translocation process are carried out by an enzyme producing a homopolymer [80].

**Figure 2:**
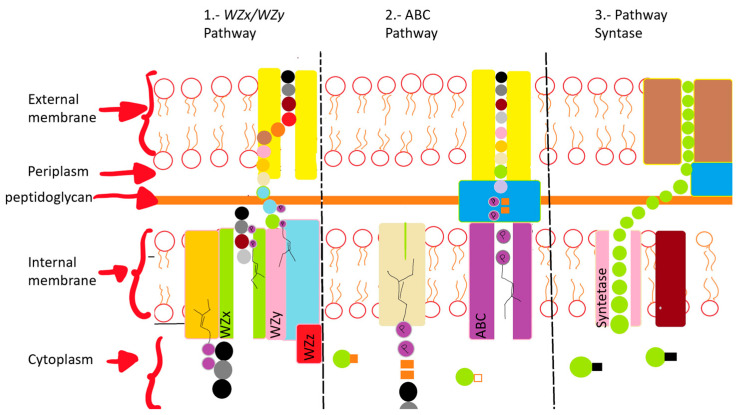
Diagrammatic description of three EPS production pathways. (1) *Wzx/Wzy* pathway formed by several glycosyltransferases; the translocation to the periplasm is performed with the Wzx flippase; the polymerization is carried out in the polymerase; *Wzy* Proteins carry out transport across the membranes. (2) *ABC* transporter-dependent pathway assembles the polysaccharide chain, which is then transported through membranes and the cell wall with the participation of proteins. (3) Synthase pathway carries out polymerization and transport through the synthase complex.

The pathways are shown in Figure 2.

In some cases, such as that of *Vibrio alginolyticus*, it has been shown that the production of EPSs takes place once their growth reaches the stationary phase, and their production behavior is non-linear because they are produced by stress; therefore, they will have a production that is seen in different stages [92].

EPSs can be recovered by precipitation with alcohol and by chromatography. The steps for their recovery are simple: removal of the cells, elimination of proteins in the EPSs, precipitation, and purification. Table 5 shows some techniques used to remove the proteins, and Table 6 shows some precipitation techniques [92].

As aforementioned, EPSs of marine origin are of great interest at the scientific and industrial levels, due to the properties shown, which are derived from the differences in the chemical structure compared to EPSs of terrestrial origin, e.g., the high content of uronic acid and sulfate groups increases their antioxidant activity, as well as their bioadsorption properties [71].

Marine EPSs have some limitations for their application, such as the low amounts of production of some microorganisms [71], the mutations or modifications that microorganisms may have at the DNA or metabolic levels, which can stop the production of EPSs [91], and the material of the container (flask, Petri dish, tube) in which they are found can influence their production [96]. 

The path for the use of marine EPSs on a large scale is still long and more research is required because the results are mostly still preliminary, but interest has been gained due to their different and superior properties compared to their terrestrial counterparts, and for being treated with exopolysaccharides that are not derived from petroleum and are biodegradable, criteria that are currently important for sustainable industries.

## 5. Enzymes 

Enzymes are biological catalysts that carry out chemical reactions for cells. The most important aspects of enzymes are stereochemical properties, specificity, and affinity for certain substrates, among others. Currently, 3000 enzymes have been identified, of which 65% have uses in the textile, paper, and starch industries, and 25% in the food industry, with the detergent industry using 40% of enzymes worldwide. The marker for industrial enzymes was estimated to reach the value of 3.3 billion USD in 2010 and 10.5 billion USD by 2024, with a compound annual growth rate of 5.7% for the period of 2018 to 2024 [97]. In addition to the uses in these areas, they can be found in the environmental field as biomarkers [98] and bioremediation [99], among others; in the medical field as bactericides, for medical tests, in medical treatments, etc. [100].

Marine enzymatic biotechnology is relevant due to the properties that marine enzymes have, since these microorganisms need a unique molecular mechanism to survive, which they obtain by adapting to their ecological niche, and by evolution. Marine enzymes offer among their main characteristics: hyperthermostability, halophycity, barophicity, adaptability to cold [101,102], chemoselectivity, regioselectivity, degradability of recalcitrant molecules, stereoselectivity, and tolerance to solvents, which is almost always shown in halophilic enzymes. In addition to this, they are more stable than enzymes obtained from animals and plants [103,104,105].

The search for enzymes can be carried out via two methods. The first method is through the cultivation of microorganisms and enzymatic selection with special culture media, and the second is through metagenomics. This is usually simple, but it is more complicated to obtain information since non-culturable microorganisms are found. The second method is through the sequence of the gene that codes for the enzyme which can be obtained and placed in another microorganism to produce with the same properties [105].

Some of the most important marine enzymes are:

Hydrolases: Those such as cellulases, hemicellulases, ligninases, and xylanases. Hydrolases from unconventional sources have been sought for the degradation of biomass and its polymeric complexes. Among the enzymes responsible for breaking down polysaccharides to obtain smaller compounds, the most valuable is xylanase, whose main use is in the paper industry where it is used to remove xylan in water and thus avoid contamination; some producing microorganisms are *Halomonas meridian*, *Microbulbifer hydrolyticus*, *Bacillus berkelegi Vibrio*, among others [102,106,107,108,109].

Xylanase: This can be used to obtain high value-added products such as xylitol, it helps make lignin soluble and less chlorine is used in the paper industry, and it can degrade polysaccharides in juices or beer, helping in clarification, etc. Some examples of producing microorganisms are *Saccharophagus degradans*, *Microbulbifer* sp., *Pantoea ananatis*, and *Bacillus aquimaris.* The latter has been shown to have the ability to tolerate solvents, which is why it is considered ideal for industrial uses, in addition to its optimum temperature of 30 °C instead of 50 °C, which shows a reduction in operating costs [77,103,107].

Proteases: These represent 60% of industrial sales worldwide and are enzymes capable of breaking down different proteins, and are generally classified based on three criteria, location with respect to the cell, site of attack on the protein, and structural similarity [59]. Some examples of producing microorganisms are *Bacillus megaterium*, *Bacillus amyloliquefaciens*, *Bacillus licheniformis*, *Bacillus circulans*, *Psychrobacter* which produces 30 different proteases, including some alkaline proteases, such as *Bacillus mojavensis*, which are compatible with surfactants and alkalis, making them good candidates for use in detergents [101,108,110].

Lipases: These break down fats and oils, releasing fatty acids and glycerol. Lipases were discovered in 1925 in *Penicillium oxalicum*, *Moraxella* which has a lipase capable of functioning at 3 °C, *Bacillus pumilus* which produces a lipase that maintains 85% activity at 5 °C, currently, a commercial enzyme obtained from polar marine microorganisms is Lipozyme, obtained from *Candida antartica* and marketed by Novozymes A/S (Denmark) [107,111].

Some marine enzymes that are already on the market are Stainzyme^®^ manufactured by Novozymes (Bagsvaerd, Denmark) which is active at temperatures below 20 °C, Preferenz^®^ manufactured by DuPont (Wilmington, DE, USA) which is active at 16 °C (these two are hydrolases), and Fuelzyme^®^ which is an α-amylase produced and marketed by Verenium Corporation (now part of BASF, Ludwigshafen, Germany), which exhibits a diverse operating range of temperatures and pH, compared to other commercially available amylases, and also requires less Ca^2+^ for stability, which facilitates further processing and lowers cost [107,110,111].

Chitinases: These degrade chitin, which is a polymer with important biotechnological properties such as improving the immune system, improving digestion, and eliminating toxins from the body. It is the second most common polysaccharide after cellulose, and its degradation into smaller molecules allows its use as fertilizer, fungicide, antiparasitic, and insecticide, among others. Some producing marine microorganisms are *Vibrio fluvialis*, *Clostridium sp*, *Vibrio parahaemolyticus*, *Vibrio mimicus*, *Chromobacterium*, *Vibrio alginolyticus*, *Pseudomonas*, *Serratia*, *Vibrio algynoliticus*, *Listonella anguillarum*, *and Vibrio harveyi*. An enzyme from a marine strain of *Alteromonas* sp has been shown to have good enzymatic activity at 0 °C, while *Streptomyces champavatii* produces enzymes with activity at acidic pHs [105,107,112].

Amylases: They are enzymes that break down starch and glycogen to convert them into sugars, they belong to the family of glycosyl hydrolases, they produce about 30 reactions and have 35 subfamilies, and they are used in the food, textile, and paper industries. Some examples of marine microorganisms that produce these enzymes are *Rhodothermos marinus*, *Aurebasidium pulluans*, *Bacillus licheniformis*, *Micrococcus sp*, *Vibrio alginolyticus*, *Halobacillus*, *Alteromonas haloplanktis* (the latter produces the enzyme at 4 °C and its activity is 7 times higher than that of thermophilic enzymes), *Thermococcus hydrothermalis* has catalytic activity at 95 °C and pH between 4 and 8 and some authors [107,111,113] reported a new α-amylase, which was obtained from a metagenomic library, whose optimal activity is at 50 °C and it retains its activity at 0 °C [113].

Alginate lyases: The potential of these enzymes is mainly in the degradation of marine biomass, specifically in the case of brown algae, which are the most abundant and can cause environmental problems. They also have uses in the medical, food, and environmental fields, since it has been reported that they are capable of serving as antibacterial because they degrade the biofilms of some microorganisms such as *Pseudomonas aeruginosa*, making them more sensitive to other treatments. *Bacillus velezensis*, *Pseudoalteromonas*, and *Vibrio alginolyticus* are some of the microorganisms capable of producing these enzymes [114,115,116,117].

Agarase: Agar is a polysaccharide commonly found in algae and is used in the cosmetics and food industries. These enzymes can be divided into three main groups: those that degrade agar, those that liquefy agar, and those that only soften agar, some of the first isolated microorganisms capable of producing this enzyme were *Pseudomonas galatica*, some species of *Vibrio*, and *Bacillus* [107].

Carrageenases: Carrageenan is used in food. About 80% of use in the food industry are sulfated polysaccharides, and carrageenases break them into smaller molecules (oligosaccharides) with antiviral, anticoagulant, and antitumor activities, among others. Some marine microorganisms that produce these enzymes are *Pseudomonas*, *Cythopagia*, *Alteromonas atlantica*, and *Alteromonas carraageenovora* [107].

Psychrophilic enzymes: Among the marine enzymes, the psychrophilic enzymes could be said to be one of the most interesting groups, due to the low temperatures they require to carry out their reactions. The Antarctic environment is characterized by challenging conditions, in terms of salinity and temperature, both of which affect the viscosity of the water, making it difficult for chemical reactions to take place, therefore it can be assumed that the density of the microorganism decreases under these conditions, but in reality, the microbial viability is maintained. The mechanism of action of psychrophilic enzymes is to decrease the reaction enthalpy [95].

Fibrinolytic enzymes: These have been studied due to their use in the medical field to combat hrombosis, where they have been obtained from marine strains of *Bacillus vallismortis*, *Marinobacter aquaeoli*, *Bacilus subtilis*, *Bacilus velezensis*, among others, whose optimum temperature of enzymatic activity ranges from 33 to 60 °C depending on the microorganism [118,119].

Antimicrobial enzymes: These are endowed with bactericidal properties, their methods of action are the degradation of molecules such as DNA, polysaccharides, and proteins, they can lead to the inhibition of enzymes, and prevent the formation of biofilms that serve as protection for bacteria. Some bacteria with these properties include *Pseudoalteromonadaceae*, and *Vibrionaceae* [105,119,120].

Enzymes are involved in the biogeochemical cycles of carbon, nitrogen, and halogens, among others [120,121], so their presence and that of the microorganisms that produce them depend on the available substrates. Various articles have reported enzymatic activities in different areas and seasons of the year, obtaining diverse results in terms of enzyme production. Arnosti [122] tested six different polysaccharide hydrolase activities in seven seasons in the eastern Pacific, Gulf of Mexico, Skaggerak (Denmark), and the Arctic. Of these activities, only one (laminarinase) was measurable at all sites as laminarin has been shown to be important in biogeochemical cycling, Also, was observed that the enzymatic activities have an important variation in one day since the activities vary 2 or 3 times per day. Arnosti [122] detected variations in the seasons, with the activity of lipases, B-glucosidases, and aminopeptidases being higher in summer with the arrival of tourists [122,123,124,125].

Other enzymatic activities: These are L-asparaginase whose use is for cancer treatments and reduction of acrylamide in food [85], phosphatases used in molecular biology to prevent DNA from folding into a helix, uracil DNA glycosidases to eliminate uracil from DNA [110], AHL lactonase used in aquaculture to eliminate pathogens (biological control) [85], ureases for blood kits and heavy metal detection [126], glucosidases for diabetes control as it removes the absorption of sucrose [127]. 

Marine enzymes have great potential for various applications due to their unique characteristics, since the changes they have range from sulfation because they are marine, have different structures, have more specific activities, have greater tolerance to different pHs and temperatures, and require fewer cofactors, among others. Marine enzymatic biotechnology is still in its infancy and its potential is great, with the help of metagenomics, enzymes with better characteristics can be obtained despite being in microorganisms that are uncultivable.

Table 7 shows some enzymes obtained from marine origin and their possible uses.

## 6. Metagenomics

Metagenomics has offered several benefits, it allows to sample microbial communities directly from their natural habitats without the need of isolation and cultivation of individual microorganisms which leads to lower time in the research for new bioproducts and the increase in knowledge of microorganisms since only can be isolated 0.001–1% of them [133], also it can help to have a biomonitoring of the microorganisms and the factors that can affect the microbial niche [134], and can reveal degradation and utilization features across free living and host associated marine microbiomes as shown by Raimundo et al. [135], were they suggest that the use dictates the processing of chitin in marine niches and supports the hypothesis that this interactions could facilitate the coexistence of chitin utilizers in marine invertebrate microbiomes, thanks to that interactions they can also detect if a dysbiosis is happening in the microbiome.

In the case of enzyme detection in marine environments, marine sediments have been used frequently, where it has been shown that there is a greater microbial diversity and that they are even more phylogenetically diverse than other environmental types including the soil [136,137], for pigments metagenomics also has wide use to explore the diversity of pigments that can be found in the deep sea at different depths, as shown by Cho et al. [138], they indicate depth dependent variations in carotenoid biosynthesis pathways, for example, The astaxanthin as it is mentioned above helps against UV radiation, but in the case of the microorganisms studied, variations on it are observed according to the depth.

Whole genome sequence mining has been used to discover biosynthetic pathways for novel biproducts which has helped find new genes and provide better insight into the microbial genetics of different marine habitats that were previously inaccessible, but metagenomics has limitations like low resolution, bias classification of short target segments, and false functional confirmation [133].

## 7. Conclusions

The 21st century has been the century of the ocean because it has proved to be a source of relevant biological and biotechnological material, that provides a new source of novel resources with different characteristics for human beings. Products of marine origin have become increasingly important due to their applications. However, to date, the exploration of these marine resources has been minimal due to technological restrictions, and the ocean presents an opportunity to tap into this potential, discovering new organisms, enzymes, and biochemical pathways that could have significant industrial applications like the fibrinolytic enzymes produced by *Marinobacter aquaeolei*, the discovery and potential use of marine microorganisms in various industrial fields can be promoted as shown in the present review. In addition to the discovery of bioproducts, industries must adapt to the processes of these microorganisms since they can be different, but at the same time, they can use biological residues of marine origin to produce certain bioproducts such as chitinase enzymes and exopolysaccharides with special features that makes them different to their homologous.

With the help of genome technology, gene recombination, synthetic microbiology and bioinformatics, the isolation of microorganisms and bioproducts detection is developing quickly, making its use in the future more promising in emerging industries, however only 179 new bioactive compounds have been characterized [139], therefore extensive research is required in the field of bioproducts derived from marine bacteria, some of them have advanced more in their use and research but others require more technologies and adaptations for their use

## Figures and Tables

**Table 1:** Marine bacteria and their bacteriocins.

Bacteria	Bacteriocins	Inhibition and Characteristics	Weight (kDa)	Reference
*Bacillus licheniformis* BTHT8	*P. putida* FStm2	*Bacillus thuringiensis*, *S. aureus*, *E. coli*, *Enterobacter aerogenes*, *Serratia marcescens.*	32	[9]
*Lactobacillus fermentum* SBS001	*L. fermentum* SBS001	*Klebsiella oxytoca*, *P. aeruginosa*, *E. coli*, Thermostable	78	[9]
*Vibrio* spp. MMB2	BLIS vibriocin AP10	*Aeromonas hydrophila*	16	[9]
*Enterococcus faecalis*	*E. faecalis*	*S. aureus*, *Bacillus subtilis*	94	[9]
*Bacillus sonorensis* Mt93	Sonoresin	*Listeria monocytogenes*, *S. aureus*	6	[9]
*Lactobacillus delbrueckii*	Bacteriocin PSY2	*Arthrobacter* sp., *Acinetobacter* sp., *B. subtilis*, *E. coli*, *L. monocytogenes*, *P. aeruginosa*, *S. aureus*	10	[9]
*Lactobacillus casei*	Bacteriocin AP8	Thermostable, resistant to different pH’s, Used against *C. perfringens* y *L. monocytogenes*		[9]
*Lactobacillus plantarum*	Bacteriocin H5	Thermostable, works against *C. perfringens* y *L. monocytogenes*		[9]
*Vibrio harveyi* VIB 571	BLIS	*Vibrio fischeri*, *V. gazogenes*, *V. parahemolyticus*	32	[19]
*Vibrio vulnificus*	IW1	*V. cholera*	9	[19]
	BC1	*V. parahaemolyticus*	7.5	[19]
	BC2	*Vibrio* spp., *Plesiomonas shigelloides*, *E. coli*	1.35	[19]
*Aeromonas hydrophila*	BLIS	*Staphylococcus aureus*		[19]
*Pseudoalteromonas* X153	P-153	*Ichthyopathogenic vibrio*, *Staphylococcus epidermidis*, *Propionibacterium acnes*, *Propionibacterium granulosus*	280	[19]
*Enterococcus faecium* LHICA	Enterocin P	*Carnobacterium maltaromaticum*, *L. monocytogenes*, *S. aureus*		[19]
*Enterococcus faecium*	Bac ALP7 Enterocin	*L. monocytogenes*	10	[19]
*Pediococcus pentosaceus* ALP57	Bac ALP57 Pediocin	*B. subtilis*, *E. faecalis*, *L. brevis*, *L. curvatus*, *Listeria innocua*	4509	[19]
*Carnobacterium divergens* V41	Divercin V41	*L. monocytogenes*	4416	[19]
*Carnobacterium pisicola* V1	PisciocinV1a y V1b	*L. monocytogenes*	4526	[19]
*Pseudomonas putida* FStm2	*P. putida* FStm2	*B. thuringiensis*, *S. aureus*, *E. coli*, *E. aerogenes*, *S. marcesens*, *Salmonella* sp.	32	[19]
*Bacillus pumilus*	Pumiviticin	*S. typhimurium*, *Proteus vulgaris*, *M. luteus*	3.9	[20]
*Lactobacillus acidohphilus*	Sonoresin	*L. monocytogenes*, *S. aureus*, *S. enterica*, *E. faecalis*	6274	[20]
*Lactococcus lactis*	Bacteriocin PSY2	*Athrobacter* sp., *Acinetobacter* sp., *B. subtilis*, *E. coli*, *L. monocytogenes*	25	[20]

**Table 2:** Marine pigments found in marine bacteria—activities and color.

Pigment	Activity	Microorganism	Color	References
Undecilprodigiosin	Anticancer	*Streptomyces* sp.	Red	[48]
Cycloprodigiosin	immunosuppressant	*Pseudoalteromonas denitrificans*	Red	[49]
Heptyl prodigiosin	Antiplasmodial	*Proteobacteria*	Red	[50]
Prodigiosin	Antibacterial, algaecide, anticancer	*Pseudoalteromonas rubra*	Red	[51]
Astaxanthin	Antioxidant	*Agrobacterium aurantiacum*, *Pseudoalteromonas luteviolacea*	pink	[52,53]
Violacein	Antibiotic, anticancer	*Pseudoalteromonas* sp., *Collimonas*	violet	[54]
Phenazine	Cytotoxic	*Bacillus* sp.	yellow	[55]
Pyocyanine	Antibacterial	*P. aeruginosa*	Blue-green	[56]
Tambjamine	Anticancer, Antibiotic	*Pseudoalteromonas tunicata*, *Vibrio cholerae*	Yellow-red	[57]
Melanines	UV Protection	*Alteromonas nigrifaciens*, *Cellulophaga tyrosinoxidans*	Brown-black	[58]
Scytonemin	UV Protection	Cyanobacteria	Brown	[58]
Tryptamine	antibiotic	*Cytophaga*, *Flexibacteria*	Yellow-orange	[58]
Quinone	Antiapoptotic properties	*Roseobacter litoralis*	Yellow-orange-red-black	[41]
Indigoids		*Methylophaga aminisulfidivorans*	Purple-blue	[41]
Zeaxanthin		*Aquibacter zeaxanthinifaciens*, *Zeaxanthinibacter enoshimensis*, *Mesoflavibacter aesturarii*		[59]

**Table 3:** Classification of EPSs according to their components.

Compound	Example
Pentoses	D-arabinose, D-ribose, D-xilose
Hexoses	D-glucose, D-galactose, D-manose
Amino-sugars	D-glucosamine, D-galactosamine
Uronic acids	D-glucoranic acid, D-galacturonic acid

**Table 4:** Exopolysaccharides produced by marine bacteria and their applications.

Microorganism	Source	Description	Use	References
*Alteromonas infernus*	Deep sea water, hydrothermal vent	Two EPS one with uronic acid and the other heteropolysaccharide with uronic acids	Sewage treatment	[81,90,91]
*Vibrio* sp.	Deep sea and invertebrates	Heteropolysaccharide, with uronic acids, and amino sugars	Anticoagulant, antiviral	[90]
*Pseudoalteromonas* sp.	Invertebrates (Annelids), deep sea	Sulfated heteropolysaccharide, high content of uronic acids and pyruvate	Wastewater treatment and bioremediation	[81,90]
*Alteromonas macleodii*	Marine water, deep sea	Sulfated heteropolysaccharide with a high content of uronic acids	Thickener and as a treatment for bone healing, cardiovascular diseases, bioremediation, and cosmetics	[73,74,90]
*Alteromonas* sp.	Isolated from alvinellidae	Sulfated heteropolysaccharide	Anticoagulant Activity and Metal Binding	[90,92]
*Vibrio diabolicus*	Isolated from a Pompeii worm and a hydrothermal vent	Sulfated Heteropolysaccharide, rich in uronic acid	Bone regeneration (patent)	[83,91]
*Vibrio harveyi*	Shrimp	Sulfated heteropolysaccharide with uronic acid		[90]
*Haloferax mediterranei*	Mediterranean Sea	Heteropolysaccharide, rich in uronic acid	Oil recovery	[86,90]
*Hahella chejuensis*	marine sediment	Heteropolysaccharide, rich in uronic acid	Biosurfactants and detoxification of areas contaminated with oil	[90,93]
*Pseudoalteromonas*	Sea sediment, Antarctic ocean, plankton	Sulfated homopolysaccharide and heteropolysaccharide	Bioremediation, cryopreservation	[90]
*Colwellia psychrerythrea*	Marine sediments and deep water	----	Cryopreservation and degradation of hydrocarbons	[74,90]
*Halomonas* sp.	Marine sediments, seawater	Sulfated homopolysaccharide and heteropolysaccharide of Mannose	Cancer therapy	[73,88]
*Haloterrigena turkmenica*		Sulfated heteropolysaccharide with uronic acid	Emulsifier and to maintain moisture in food	[73]
*Vibrio alginolyticus*	Seafoam	Sulfated heteropolysaccharide with uronic acid and heteropolysaccharide with amino sugars	Emulsifying and with pseudoplastic properties	[80,83]
*Pseudoalteromonas*	Marine sediment	Sulfated heteropolysaccharide with uronic acid	Gelling agent	[74,84]
*Thermococcus litoralis*		Sulfated heteropolysaccharide with uronic acid and thermophilic properties	Industrial purposes	[74]
*Bacillus denitrificants*	Marine sediment	Sulfated heteropolysaccharide	Antiviral activity	[74,80]
*Halomonas ventosae*	Saline soils	Heteropolysaccharide	Emulsifier	[92]
*Enterobacter cloacae*	Marine sediments	Sulfated heteropolysaccharide	Bioadsorption of metals	[92]
*Rhodococcus erythropolis*	Pacific Ocean	Sulfated heteropolysaccharide with glucuronic acid	Emulsifying	[92]
*Vibrio furnissi*	Estuaries	Acid heteropolysaccharide	Emulsifying	[92]
*Polaribacter* sp.	*Laminaria* algae	Heteropolysaccharide with aminosugars	Antioxidant and cryopreservative	[92]
*Pseudomonas stutzeri*	Marine sediments	Heteropolysaccharide with aminosugars	Antioxidant	[92]
*Bacillus altitudinis*	Mangroves	Heteropolysaccharide with mannuronic acid	Antitumor activity	[92]
*Brevundimonas subvibroides*	Marine sediment	Heteropolysaccharide with mannuronic and sulfated acid	Anticancer activity	[92]
*Thalassospira*	Gulf of Mexico water	Protein-rich EPS	Hydrocarbon degradation	[92]
*Alcaligenes*	Marine water	Sulfated heteropolysaccharide	Antibacterial	[92]
*Bacillus xiamenensis*	Deep sea water, the intestinal tract of fish	Sulfated heteropolysaccharide	Bioremediation of metals	[92]
*Idiomarina fontislapidosi*	Hypersaline water	Anionic heteropolysaccharide	Bioremediation of metals	[92]
*Salipiger mucosus*	Mediterranean Sea	Sulfated heteropolysaccharide with pyruvic acid	Bioremediation of metals	[92]
*Zunongwangia profunda*	Deep sea sediment		Antioxidant activity	[80]

**Table 5:** Techniques to remove proteins from EPSs.

Technique	Procedure
TCA (trichloroacetic acid)	4–20% with stirring TCA with ethanol precipitation
Hydrochloric acid	12 M hydrochloric acid and temperature of 70 °C
Enzymatic	Proteases
Organic solvent	Chloroform and n-butanol 4:1

**Table 6:** EPS recovery techniques [93].

Technique	
Solvents	Acetone, ethanol, methanol, isopropanol
Salts	NaCl, KCl, MgSO_2_, MgO, CaCl_2_
Acids	HCl for anionic EPS

**Table 7:** Enzymes of marine origin, source, and applications.

Enzyme	Microorganism	Uses	References
Glucanase	*Streptomycete*, *Photobacterium panuliri*, *Pantoea dispersa*, *Shewanella* sp.	Cattle raising.	[4,128,129]
Agarase	*Vibrio* sp., *Catenovolum agarivorans*, *Microbulbifer maritimus*, *Aeromonas* sp., *Microbulbifer elongatus*, *Microbulbifer thermotolerans*	Food industry, pharmacy, and chemical industry.	[129]
Alginate lyases	*Saccharophagus degradans*, *Vibrio* sp., *Vibrio alginlyticus*	Biochemicals and biofuels.	[129]
Thananases	*Phormidium valderianum*	Food industry, gallic acid production	[129]
Chitinase	*Athrobacter* sp., *Streptomyces* sp., *Rhodotermus marinus*, *Paenibacillus* sp., *Pantoea dispersa*, *Bauveria bassiana*, *Paenibacillus sabina*, *Alcaligenes faecalis*, *Alteromonas* sp., *Paenibacillus baregonltzii*, *Vibrio alginolyticus*	Anticancer, antimicrobial and hemolytic activity, to produce prebiotics that can be used in fish and shrimp.	[129]
Carrageenan	*Pseudomonas carrageenovora*, *Cytophaga* sp., *Pseudomonas elongata*, *Pseudoalteromonas porphyrae*, *Microbulbifer thermotolerans*, *Cellulosimicrobium cellulans*, *Zobellia* sp., *Vibrio* sp., *Paenibacillus* sp., *Verticillium lecanii*, *Rhodopiruella baltica*	Food and medicine, antibacterial and antitumor activity	[129]
DNA glycosylase	Marine Strain BMTU3346	Bactericide	[129]
Xylanases	*Pseudoalteromonas haloplanktis*, *Glaciecola mesophila*, *Flavobacterium frigidarium*	Biorefineries, bioremediation, paper industry, food industry, paper industry	[129]
Esterases	*Vibrio* sp., *Plexaura homomalla*, *Pseudoalteromonas haloplanktis*, *Bacillus licheniformis*, *Staphylothermus*, *Pyrodictium* sp. *Archaeglobus* sp., *Teredinibacter* sp., *Sulfolobus* sp., *Vibrio fischeri* *Pelagibacterium halotolerans*	Reduction of undesirable compounds in detergents and removal of fatty Acids through their decomposition	[129]
L-glutaminase	*Vibrio costicola*, *Bacillus velezensis* *Providencia* sp., *Streptomyces olivochromogenes*, *Halomonas meridiana*, *Alcaligenes faecalis* *Kosakonia radiciantans*, *Vibrio axureus*, *Brevundimonas diminuta* *Pseudomonas aeruginosa*, *Streptomyces rimosus*	Anticancer properties	[4,129,130,131]
Proteases	*Pseudomonas* sp., *Aeropyrum pernix*, *Vibrio harveyi* *Bacillus clausii*, *Bacillus subtilis*, *Hyphomonas jannaschiana*, *Pantoea dispersa*, *Teredinobacter turnirae*, *Engydontium álbum* *Pyrococcus furiosus*, *Bacillus cereus*	Industrial applications, food, and detergent industry. Bactericide	[4,129,130]
Glucosidase	*Microbulbifer thermotolerans*, *Bacillus amyloliquefaciens*, *Alteromonas* sp., *Acinetobacter*, *Arthrobacter*, *Enterobacter* *Micrococcus*, *Bacillus megaterium*, *Vibrio parahaemolyticus*, *Vibrio alginolyticus*, *Leucosporodium antarticum*	Medicine	[4,110,128,130]
β-galactosidase	*Arthrobacter* sp., *Pseudoaletromonas haloplanktis*, *Enterobacter ludwigii*	Use in the dairy industry for the elimination of lactose.	[110,128]
Cellulase	*Martelella mediterránea*, *Pseudoalteromonas haloplanktis*, *Paenibacillus* sp., *Pseudoalteromonas* sp., *Bacillus* sp. *Streptomyces variabilis*, *Teredinobacter turnirae*, *Marinobacter* sp., *Rhodotermus marinus*, *Kocuria rosea*, *Stenotrophomonas maltophilia*, *Mesorhizobium*		[4,128,129,130]
Lipase	*Pseudoalteromonas haloplanktis*, *Bacillus* sp., *Brevibacterium* sp. *Geobaciullus thermodenitrificants*, *Mycobacterium tuberculosis*, *Polaromonas vacuolata*, *Psychrobacter*		[110,128]
α-amilase	*Nocardiosis* sp., *Vibrio alginolitycus*, *Aureobasidium pulullans*, *Halobacterium salinarum*, *Alteromonas haloplanctis*, *Pseudoalteromonas* sp., *Luteymonas abyssi*, *Bacillus amyloliquefaciens*, *Bacillus acidicola*, *Alicyclobacillus acidocaldarius*, *Geobacillus thermoleovorans*		[85,128,132]
CMCase	*Kocuria rosea*, *Stenotrophomonas maltophila*, *Mesorhizobium*		[4]
Alcohol dehydrogenase	*Flavobacterium frigidimaris*, *Moraxella* sp.		[110,128]
N-acetyl transferase	*Rhodococcus* sp.	Herbicide detoxification	[129]
Polymerase	*Thermotoga marítima*, *Thermotoga neapolitana*, *Pyrococcus furiosus*	Medice (PCR)	[129]

## Data Availability

Not applicable.
